# Identification of the Photoreactive Species of Protonated *N*-Nitrosopiperidine in Acid Medium: A CASPT2 and DFT Study

**DOI:** 10.1021/acs.jpca.3c06477

**Published:** 2023-11-10

**Authors:** Juan Soto

**Affiliations:** Department of Physical Chemistry, Faculty of Science, University of Málaga, Malaga 29071, Spain

## Abstract

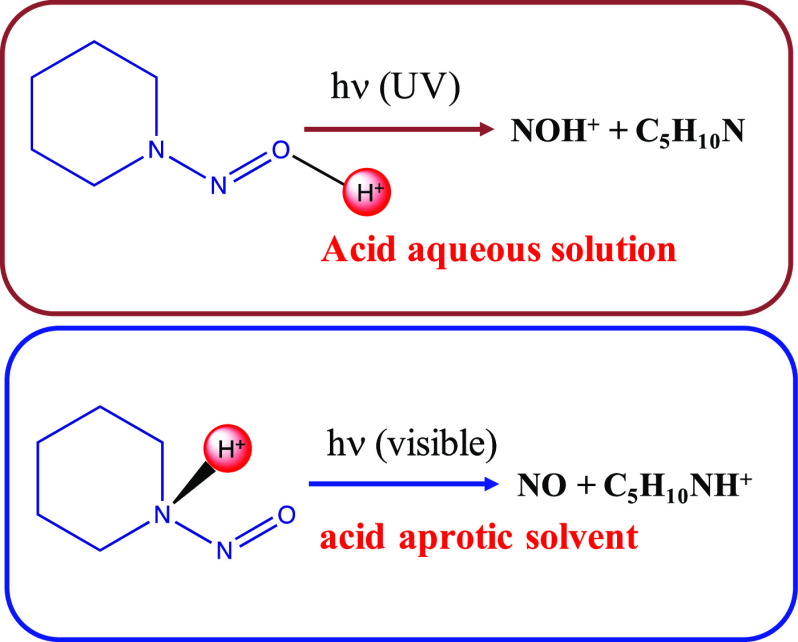

In this work, we have studied the initial reaction step after photoexcitation of protonated *N*-nitrosopiperidine both in the gas and condensed phases. To achieve this end, we have applied the CASPT2 and MP2 wave function methods and the density functional theory approach. It is found that the site of protonation of *N*-nitrosopiperidine in acid medium depends on the solvent: protonation occurs at the oxygen atom in protic solvents, while in aprotic solvents, the proton is bonded at the *N*-atom of the amine moiety. Furthermore, protonation at such an *N*-atom is the unique protonated species that absorbs in the visible range and directly dissociates into aminium radical cation and nitric oxide.

## 1 Introduction

**Scheme 1:**
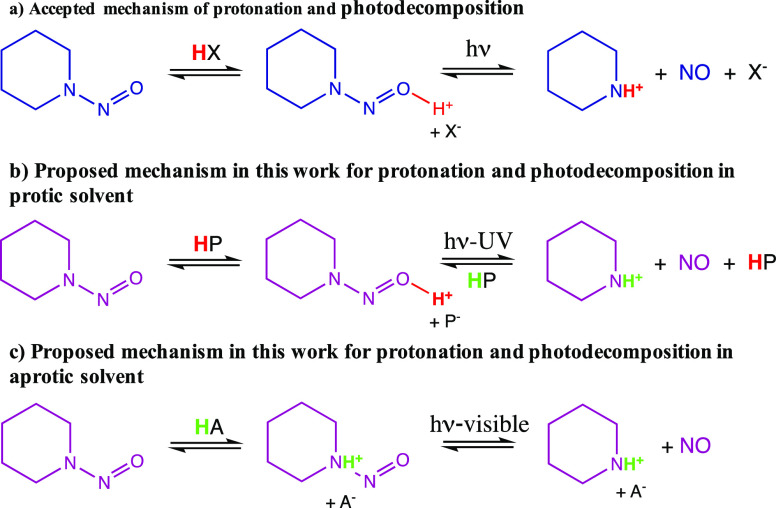
Mechanisms of Protonation and Photodecomposition of *N*-Nitrosopiperidine

Generation of nitrogen-centered radicals from *N*-nitrosamines has important synthetic applications such as functionalization of unsaturated compounds in which new C–N bonds are formed.^1−3^ Production of such radical species under mild, safe, and operationally simple working conditions is of paramount importance. In this context, recently, Patil et al.^2,3^ have nicely reported for the first time the generation of aminium radical cation from *N*-nitrosoalkylamines^2^ and *N*-nitrosopiperidines^3^ through visible-light excitation, which promotes the photoaddition reaction to alkenes and alkynes in optimal conditions. As pointed out by Patil et al.,^2^ the photoaddition of *N*-nitrosamines to alkenes was originally initiated by the group of Chow.^4−7^ Previously, it was established that the association of the NNO moiety of nitrosamines with metal ion or acid occurs at the oxygen atom.^8,9^ Therefore, since then, it is accepted that the photochemical generation of the aminium radical cation in acid medium under UV or visible irradiation arises from the protonated nitrosamine at the oxygen atom (R_2_N–N**OH**^**+**^).^3,7^ That is, UV–visible light triggers the homolytic cleavage of the N–N bond and proton migration to generate the aminium radical cation (Scheme 1a).

In this work, we propose that the nature of the solvent plays an important role in the mechanism of the photochemistry of the title molecule and related compounds. The nature of the solvent (protic or aprotic) determines the site of protonation of the nitrosamine. In protic solvents, the dominant species is R_2_N-NO**H**^**+**^, while in aprotic solvent, the only cationic species formed is R_2_N(**H**^**+**^)-NO (Scheme 1b,c). In consequence, the photoexcitation properties and photochemistry of the protonated species change with the solvent type. These assertions are based on quantum chemical calculations (CASPT2, MP2, and density functional theory (DFT)) that we will describe in the next paragraphs and whose computational details are given at the end of this manuscript.

## 2 Results and Discussion

### 2.1 Protonated Isomers of *N*-Nitrosopiperidines (Noncomplexed) CASPT2 Calculations

**Scheme 2:**
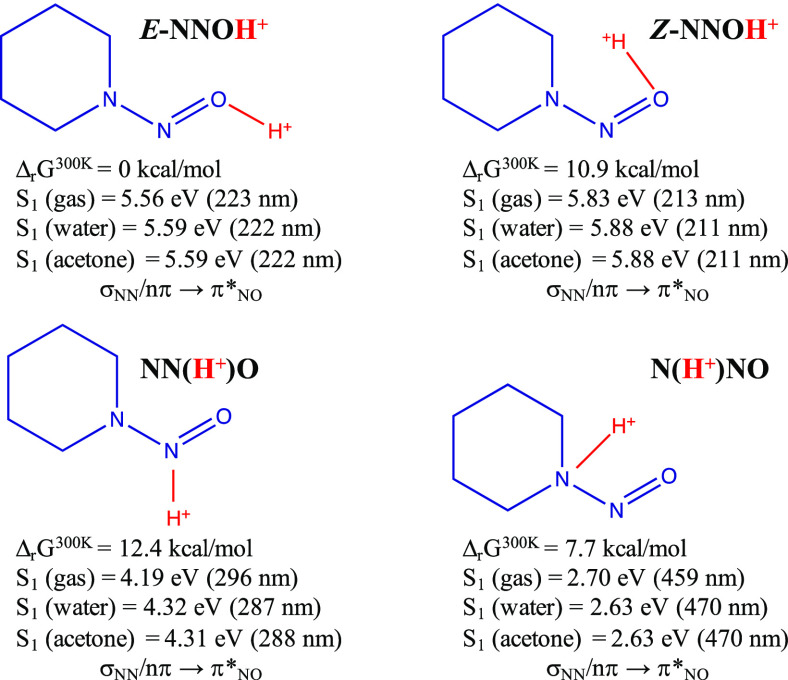
Energetics of Protonated Isomers of *N*-Nitrosopiperidines CASPT2 calculations.

**Figure 1:**
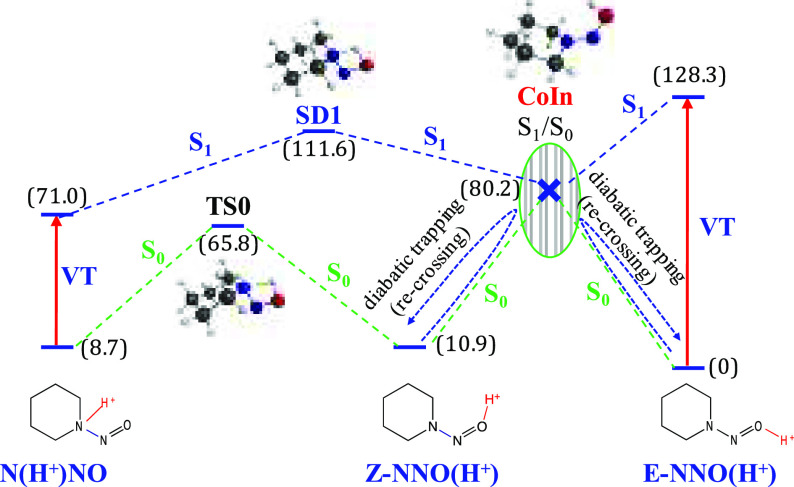
Schematic representation of the energy levels for the reaction paths on the S_1_ and S_0_ surfaces: (a) path on the S_1_ surface connecting N(H^+^)NO and *E*-NNO(H^+^)/*Z*-NNO(H^+^); (b) path on the S_0_ surface connecting N(H^+^)NO and *Z*-NNO(H^+^). CoIn: S_1_/S_0_ conical intersection. VT: MS-CASPT2 S_0_ → S_1_ vertical excitation. In parentheses: MS-CASPT2 relative energies.

The calculations presented in this subsection (Scheme 2) correspond to the protonated species (noncomplexed) both in the gas phase and solution (water and acetone). Scheme 2 shows their structures plus the relative Gibbs free energies of the four protonated compounds, the excitation energies to the first excited state (S_1_) of each compound and the character of S_1_. Table 1 reports the assignment of the MS-CASPT2 electronic transitions of neutral and protonated *N*-nitrosopiperidines included in Scheme 2. The molecular orbitals that describe the active space of each isomer are listed in Figure S1, which, in turn, correspond to the labels given in Table 1. The most remarkable feature of Table 1 is the character of the first excited state for all of the compounds, which is of the same character for all of them, that is, (σ_NN_*/n*π) → (π*_NO_) excitation. In accordance with the data presented in Scheme 2 and Table 1, two observations must be highlighted: (i) only the aminium radical 
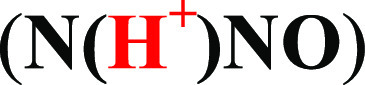
 absorbs in the visible range of the spectrum; (ii) according to the values of the relative energies given in Scheme 2, the 
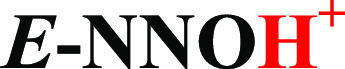
 isomer would be the majority species among the protonated species, which absorbs in the UV region. However, it must be noted that the protonated isomers are not interconvertible due to the topologies of the S_0_ and S_1_ potential energy surfaces that forbid/hinder *E***-NNOH**^**+**^ ↔ **N(H**^**+**^**)NO** isomerization. In a recent study on *N*-nitrosodimethylamine,^10^ we demonstrated that a S_1_/S_0_ crossing, which exists along the hypothetic path that would connect *E***-NNOH**^**+**^ ↔ *Z***-NNOH**^**+**^ ↔ **N(H**^**+**^**)NO**, excludes such an isomerization due to the diabatic trapping effect.^11−21^ Since we have been able to find the analogous conical intersection for *N*-nitrosopiperidine (Figure 1), we must discard the population of the different protonated isomers of *N*-nitrosopiperidine based on the relative Gibbs free energies. Thus, the formation of one or another protonated species would be dynamically controlled and not by kinetics factors.

**Table 1:**
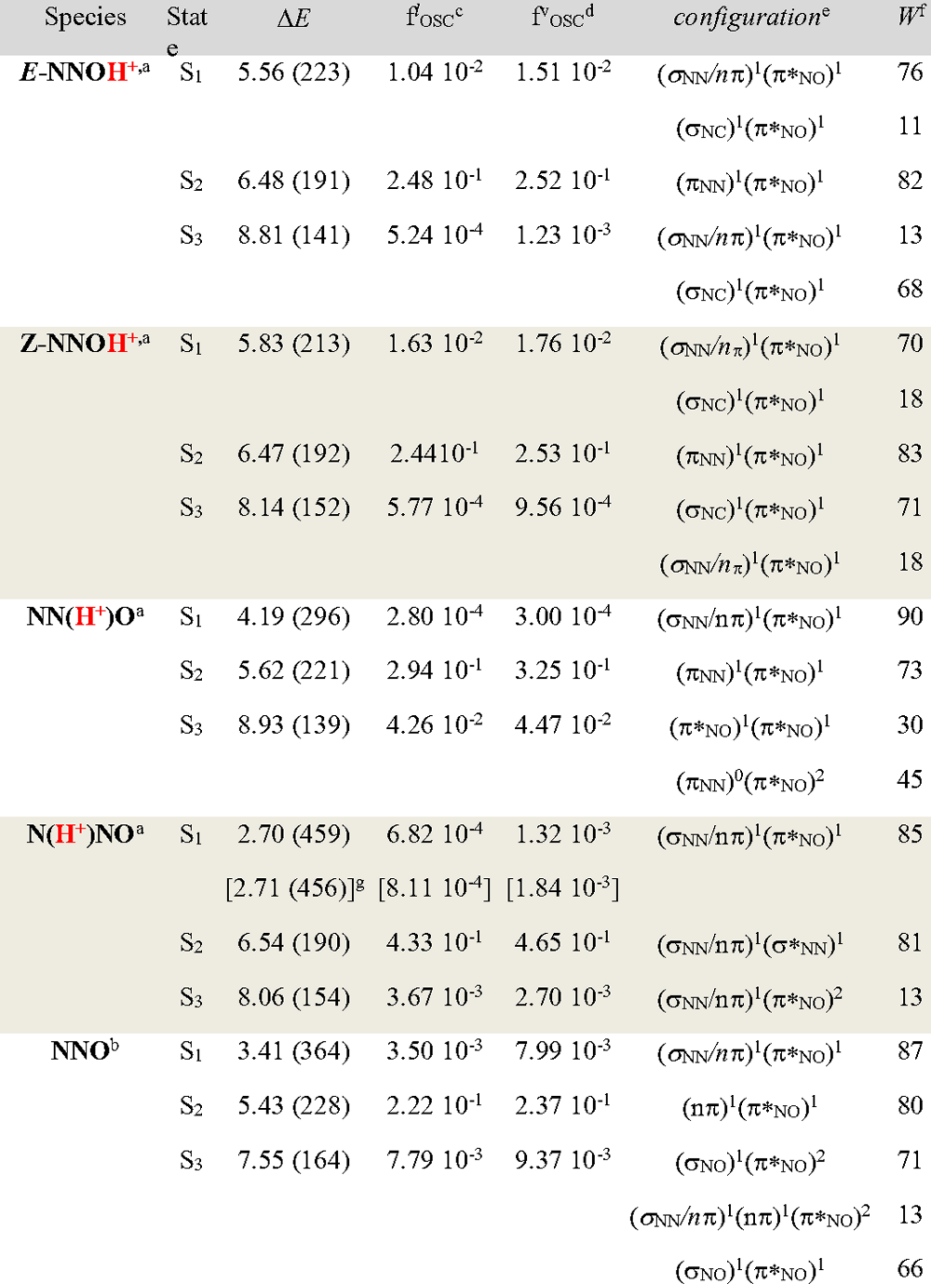
MS-CASPT2 Vertical Excitation Energies (Δ*E*) in eV (nm) of *N*-Nitrosopiperidines

a Reference wave function 1: SA2-CASSCF(16,14)/ANO-RCC (**C,N,O**[4s3p2d1f]/**H**[3s2p1d]).

b Reference wave function2: SA2-CASSCF(16,13)/ANO-RCC (**C,N,O**[4s3p2d1f]/**H**[3s2p1d]).

c Oscillator strength (length formula).

d Oscillator strength (velocity formula).

e MS-CASPT2 main electronic configurations of the excited states are referred to as the ground state configuration.

f Weight of the configuration in %. Only contributions greater than 10% are included.

g In square brackets: values including the solvent effect (PCM model) at the PCM optimized geometry.

To be specific, along the reaction path that would hypothetically connect *E***-NNO(H**^**+**^**)** ↔ *Z***-NNO(H**^**+**^**)** ↔ **N(H**^**+**^**)NO** on the S_0_ or S_1_ potential energy surfaces (Figure 1), we have localized three minima (N(H^+^)NO, *Z*-NNO(H)^+^, *E*-NNO(H)^+^), and three critical points: a transition state (**TS0**) that connects *Z***-NNO(H**^**+**^**)** with **N(H**^**+**^**)NO** on the S_0_ ground state; a saddle point (**SD1**) that connects **N(H**^**+**^**)NO** with a S_1_/S_0_ conical intersection (**CoIn**), which is the third critical point. It must be remarked the relevancy of the S_1_/S_0_ conical intersection (CoIn), as was noted in the previous paragraph, it forbids the *E***-NNO(H**^**+**^**)** ↔ *Z***-NNO(H**^**+**^**)** equilibrium in both directions due to the diabatic trapping effect, which forbids the *Z–E* isomerization on the ground state.

Minimum energy geometries of protonated *N*-nitrosopiperidines and the unprotonated parent molecule (N(H^+^)NO, *Z*-NNO(H)^+^, *E*-NNO(H)^+^, *E*-N(H)^+^NO, and NN(H)^+^O) have been optimized at the CASPT2 level with a CASSCF(16e,14o) reference wave function. Transition state (TS0), saddle point (SD1), and S_1_/S_0_ conical intersection (CoIn) have been optimized at the CAM-B3LYP/def2-TZVPP level. The energies of all the geometries represented in Figure 1 correspond to MS-CASPT2 calculations taking a two-state average CASSCF(16e,14o) reference wave function. The Cartesian coordinates in Å of all these structures mentioned in the previous paragraphs are given in the Supporting Information.

### 2.2 Complexed Isomers of *N*-Nitrosopiperidines

**Figure 2:**
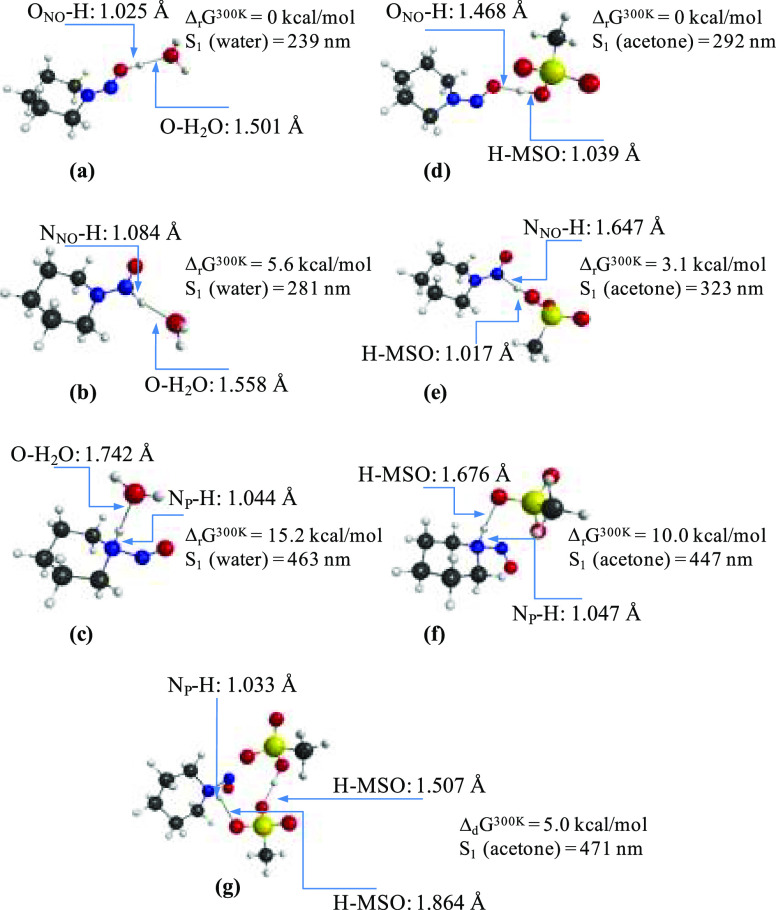
(a–g) Comparison of MP2 geometries and energetic parameters of *N*-nitrosopiperidine: X-H^+^ complexes (X: H_2_O or MsO^–^).

In the next step, we have studied the *N*-nitrosopiperidine compound complexed with hydronium cation (H_3_O^+^) or methanesulfonic acid (MsOH, used in ref (3) in solution phases, that is, water, and acetone, respectively. Figure 2 collects the MP2-optimized geometries of such complexes along with S_0_ → S_1_ excitations and relative energies. It is found that protonation of the nitrosamine in acid-aqueous solution can occur at three different sites, yielding three protonated isomers (**2a–2c**) and the protonation at the oxygen atom being the most energetically favorable binding (**2a**). Again, complex **2a** absorbs in the UV region. In contrast, the scenario is completely different in aprotic solvents; in this case, the only protonated complex of *N*-nitrosopiperidine that is formed is **2f**, the precursor of the aminium radical cation and the only complex that absorbs at the visible range. Again, the complex formed by the interaction of MsOH with the oxygen atom of *N*-nitrosopiperidine is the most stable species (**2d**). However, proton transfer from MsOH to *N*-nitrosopiperidine does not occur in **2d**. Complexes represented in Figure (**2d–2f**) have the drawback that they are energetically less favorable with respect to the separate molecules that form them. To overcome this inconvenience, we studied the complex formed by the reaction of the most stable complex **2d** with another molecule of MsOH (Figure 2g). The computed S_1_ vertical excitation energy and Gibbs free energy of dissociation of complex **2g** amount to 471 nm and 5.0 kcal/mol, respectively. Therefore, we have found a complex that is stable with respect to dissociation and absorbs in the visible region. It is worth noting at this point that the ratio *N*-nitrosopiperidine:MsH of the experiments given in ref (3) is 1:2.

### 2.3 Potential Energy Surfaces Involved in Photodissociation of Protonated *N*-Nitrosopiperidines

**Figure 3:**
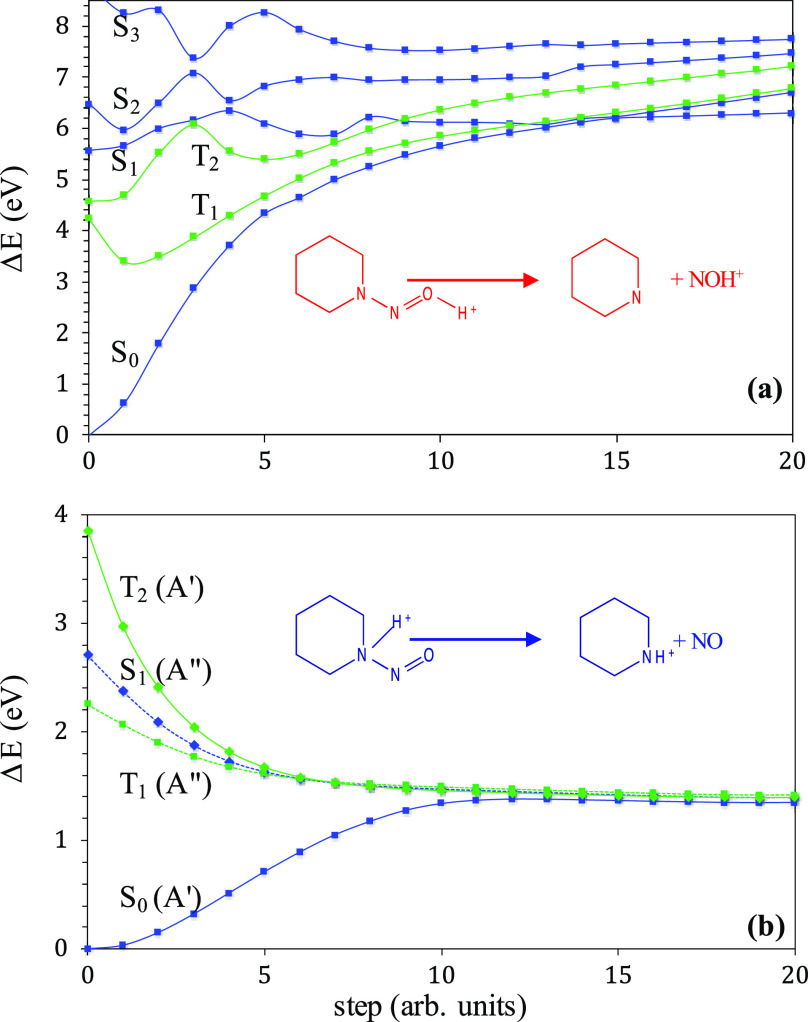
MS-CASPT2 potential energy profiles for the dissociation reaction of (a) *E*-NNOH+ into C_5_H_10_N and NOH^+^ [SA4-CASSCF] and (b) dissociation of N(H^+^)NO into the aminium radical cation and NO [SA2-CASSCF].

In this subsection, we have studied the potential energy surfaces which would lead to the N–N bond breaking of *E*-NNOH^+^ and N(H^+^)NO, respectively, after photon absorption. To achieve this end, we have applied the interpolation method at the MS-CASPT2 level with variation of the 3*N*-6 internal coordinates of the system.^22−26^ The lowest singlet and triplet potential energy surfaces leading to the dissociation of *E*-NNOH^+^ into R_2_N and NOH^+^ are represented in Figure 3a. Neither of these potential curves is dissociative and all of them are not accessible with visible light irradiation. Furthermore, excitation into the S_1_ state of *E*-NNOH^+^ directly leads the system to the S_1_/S_0_ conical intersection (Figure S2). On the other hand, Figure 3b depicts the energy profiles of the lowest singlet and triplet states for dissociation of N(H^+^)NO into aminium radical cation and nitric oxide. Clearly, the S_1_ state is dissociative. Therefore, N(H^+^)NO will immediately dissociate after visible photon absorption in the S_1_ excited state. Furthermore, dissociation in the first triplet excited state, through S_1_/T_1_ intersystem crossing, must be discarded because the electronic symmetries of both states, S_1_ and T_1_ (Figure 3b), do not allow conservation of the electron momentum, given that spin flip of the electron, in the intersystem crossing process, is always accompanied of a change of the occupied orbital by the flipping electron, which originates the well-known El-Sayed’s rules.^27−29^ On the other hand, direct population of the T_1_ state of N(H^+^)NO from S_0_ with the 2.73 eV (453 nm) wavelength is quite unlikely due to the S_0_ → T_1_ vertical excitation being out of resonance with such a wavelength, for example, in acetone the S_0_ → T_1_ vertical transition is computed (MS-CASPT2) at 2.13 eV (581 nm), while the energy of the S_0_ → S_1_ transition is computed at 2.63 eV (471 nm). At this point, it is pertinent to note that another probable channel would be S_0_ → T_1_ excitation of the nonprotonated piperidine; however, its T_1_ excited triplet state is not dissociative (Figure S3). Thus, if T_1_ state of the nonprotonated species were populated, we would expect phosphorescence emission from neutral *N*-nitrosopiperidine but not photoreaction. A phenomenon that was observed in neutral nonaqueous solutions of *N*-nitrosodimethylamine^7^ and computationally corroborated by us.^30^

## 3 Conclusions

In summary, the site of protonation of *N*-nitrosopiperidine in acid medium depends on the solvent: protonation occurs at the oxygen atom of the NNO moiety in protic solvents, while in aprotic solvents, the proton is bonded at the *N*-atom of the amine moiety. When such an N-atom is protonated, the molecule absorbs in the visible region and leads directly to NO extrusion after photon absorption. Furthermore, regardless of the nature of the solvent and the resultant protonated species, photoreaction always occurs in a singlet state.

## 4 Computational Details

We have applied the following theoretical approaches: (i) wave function calculations performed with the complete active space self-consistent field (CASSCF)^31−35^ and the multistate second-order perturbation (MS-CASPT2)^36,37^ methods as implemented in MOLCAS 8.4^38,39^; (ii) second-order Mo̷ller–Plesset theory^40^ as implemented in GAUSSIAN16^41^; (iii) DFT with the hybrid exchange-correlation CAM-B3LYP functional^42^ as implemented in ORCA.^43,44^ With respect to MS-CASPT2 results, to avoid the inclusion of intruder states in the calculations, MS-CASPT2 energies were calculated with an imaginary shift set to 0.1. Ionization potential electron affinity empirical correction has been fixed at the standard value (0.25) in all of the calculations. CASSCF calculations obtained with the state average approximation are noted as SA*n*-CASSCF, where *n* refers to the number of states of a given symmetry species.

CASPT2 and DFT calculations including the solvent effect have been performed with the polarizable continuum model (PCM).^45,46^

The ANO-RCC basis sets^47,48^ have been used in the multiconfigurational calculations of this work by applying the contraction scheme: (C,N,O)[4s3p2d1f]/(H)[3s2p1d], while the DFT calculations have been performed with the def2-TZVPP basis sets.^49,50^

One-dimensional potential energy surfaces that are represented in Figure 3 for the dissociation reactions of *E*-NNOH+ and N(H^+^)NO, are built with the linear interpolation method,^22−26^ and their electronic energies are calculated with the MS-CASPT2 approximation from the appropriated CASSCF reference wave function, a method that has been well established to study reaction mechanisms, especially, dissociation reactions.^51−57^

The vibrational frequencies, geometries, and molecular orbitals of the chemical species have been analyzed with the graphical programs MacMolplt,^58^ Gabedit,^59^ and Molden.^60^

## Supporting Information Available

Details of CASSCF orbitals included in the respective calculations; potential energy surfaces leading to dissociation of neutral *N*-nitrosopiperidine; and comparison of TD-DFT and MS-CASPT2 vertical excitation energies (PDF)
