# Supplementary material for: Identification of the Photoreactive Species of Protonated N-Nitrosopiperidine in Acid Medium: A CASPT2 and DFT Study
Source: J Phys Chem A. 2023 Nov 10;127(46):9781–6. doi: 10.1021/acs.jpca.3c06477 (PMC10683013; doi:10.1021/acs.jpca.3c06477)
Supplement: Supplementary file 1 — jp3c06477_si_001.pdf [file jp3c06477_si_001.pdf]

Supporting Information

Identification of the Photoreactive Species of Protonated *N*-Nitrosopiperidines in Acid-medium. A CASPT2 and DFT study

by

Juan Soto

[a]Department of Physical Chemistry, Faculty of Science, University of Málaga,

29071, Málaga, Spain

E-mail: soto@uma.es

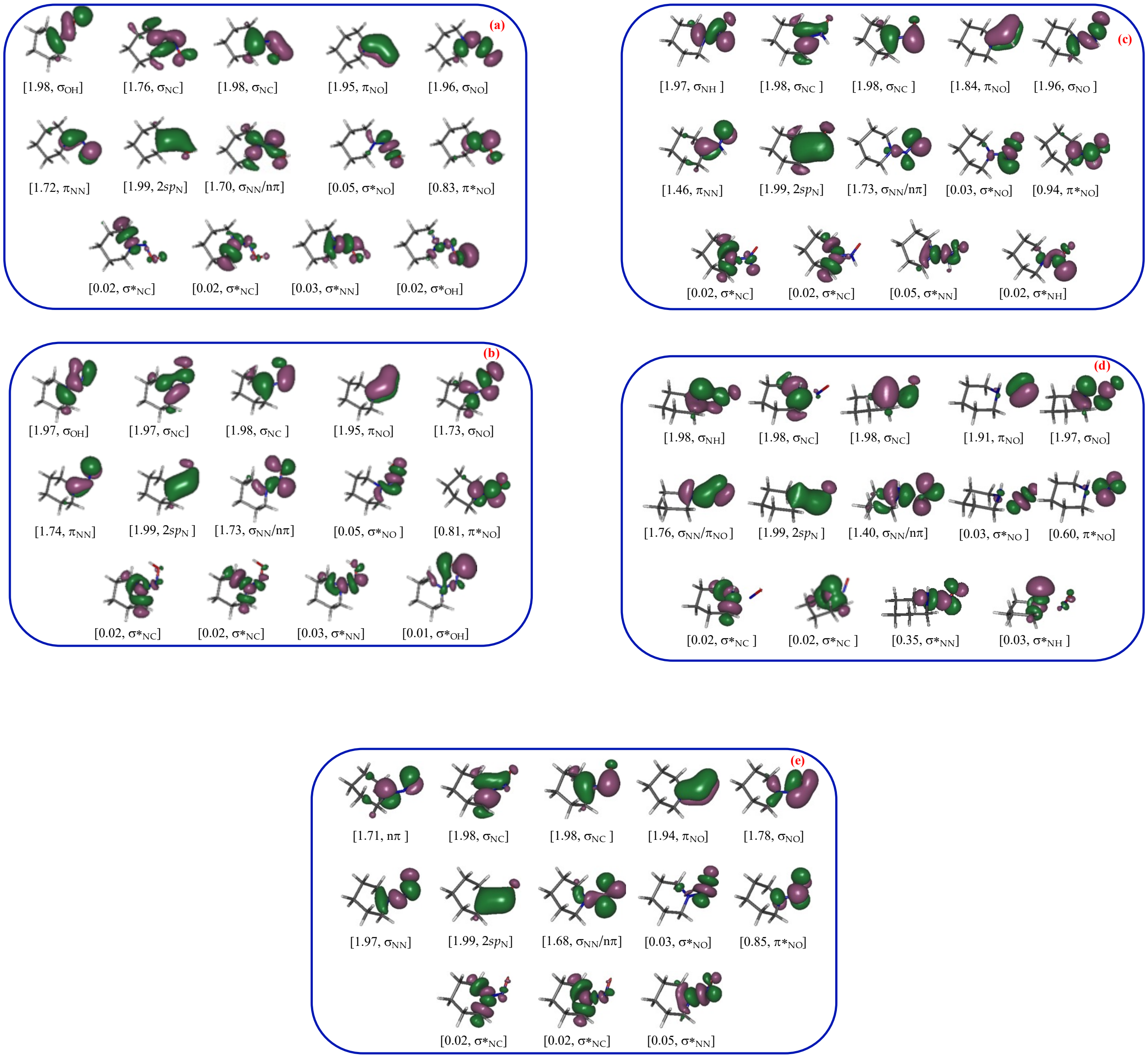

Figure S1. SA-CASSCF/ANO-RCC orbitals of (a) ***E*-NNOH$^+$**; (b) **Z-NNOH$^+$**; (c) **NN(H$^+$)O**; (d) **N(H$^+$)NO**; (e) **NNO**.

Linear Interpolations

The construction of the potential energy curves has been done with a linear interpolation method using the full space of non-redundant internal coordinates, which are built as follows: a common set of 3N-6 internal coordinates is defined for the target geometries, the reactants (R1) and the products (R2). For the dissociation case, the fragments are separated by a physically reasonable distance [Δ(R2-R1)]. Our calculations show that a value of ~4.7 Å for the dissociative bond (in this work, the N-N bond) is enough to reach the asymptotic limit of the potential energy surface (PES) with respect to dissociation of the piperidine compound into to yield NO. Difference between R2 and R1 yields an interpolation vector (ΔR) that connects reactants and products. Afterwards, ΔR is divided in *n* segments. This parameter is chosen in order to ensure a smooth convergence of the CASSCF wavefunction upon a geometrical distortion following the ΔR hyperline. Consequently, each of the divisions constitutes an interpolation step corresponding to a given nuclear conformation on ΔR. The *m*-th one is given by $R_m = R1 + (m/n)$ with $m=1,\ldots,n$. Given that our interpolation vectors result from combination of valence coordinates (internuclear distances, valence bond and dihedral angles), we cannot give a unique unit for them; therefore, we describe them as arbitrary units. Linear interpolations in internal coordinates present two main features that make them particularly appealing to these studies: (i) they are less expensive than scans with relaxation of geometry; (ii) all the points along the interpolation vector (reaction coordinate) are necessarily in a straight line within the set of defined coordinates hence providing a suitable set of coordinates for the representation of reduced dimensionality potential energy surfaces.

Thus, in accordance with the linear interpolation method, we have determined the potential energy curves that connect E-NNOH$^+$ and Z-NNOH$^+$ with the S_1/S_0 conical intersection (Figure S2), plus the dissociation processes of the electronic states of neutral *N*-nitrosopiperidine (Figure S3).

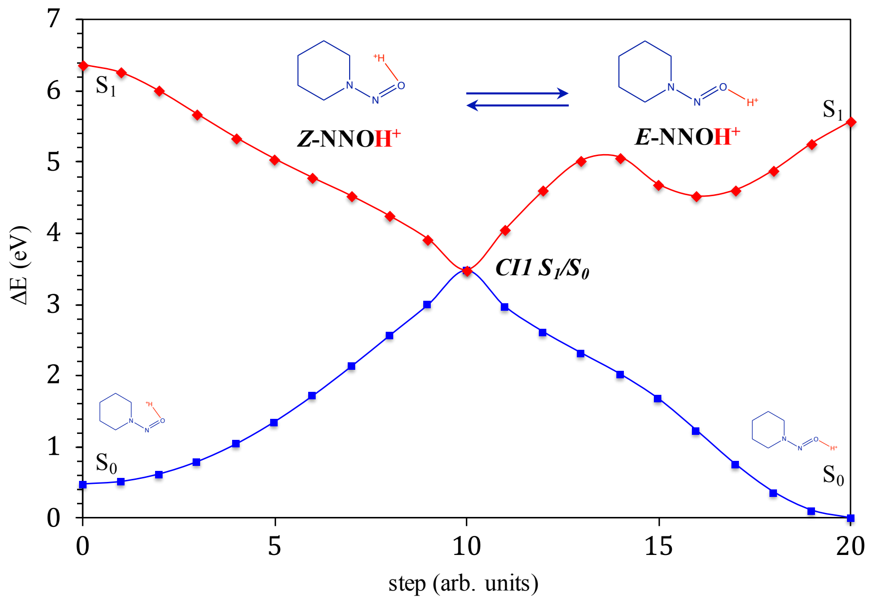

Figure S2. SA2-CASSCF(16e, 14o)/ANO-RCC potential energy curves that connect the S_0 and S_1 states of E-NNOH$^+$ and Z-NNOH$^+$ with the S_1/S_0 conical intersection. Energy of CI1: mean value of computed energies for S_1 and S_0.

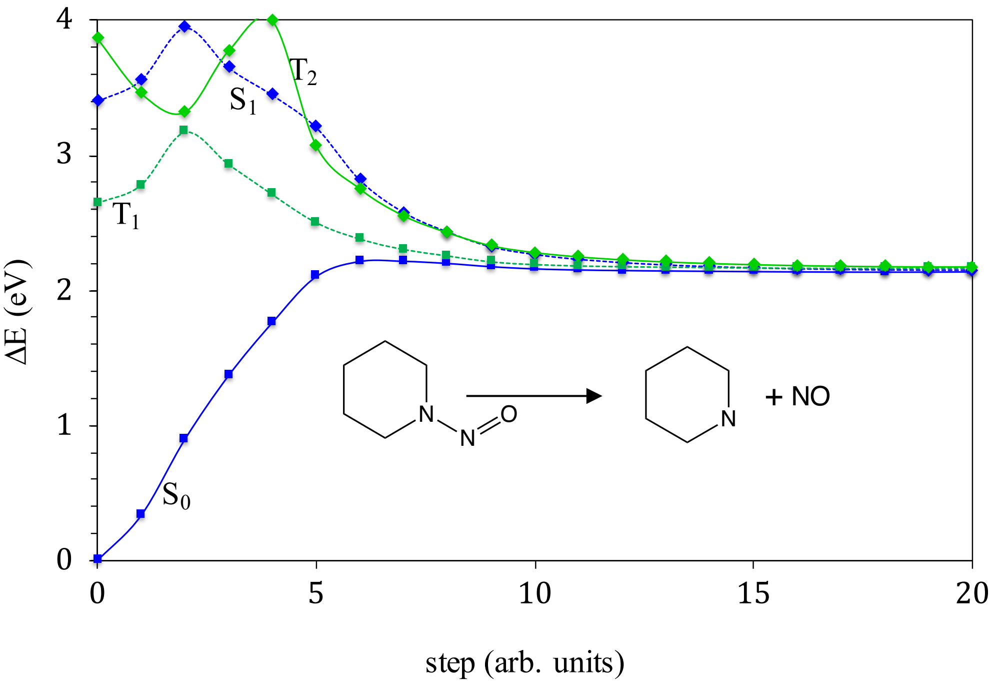

Figure S3. SA2-CASSCF(16e, 13o)/ANO-RCC potential energy curves leading to dissociation of neutral *N*-nitrosopiperidine to yield NO.

CARTESIAN COORDINATES in Å of the CASPT2/ANO-RCC optimized geometries represented in Scheme 2 in the main text. Reference wavefunction: CASSCF(16e,14o).

N(H$^+$)NO; E_h = -381.02248587 (Hartree)

N1	-0.029657	0.000000	0.039966
N2	0.017407	0.000000	1.825159
O3	1.084729	0.000000	2.218353
C4	-0.733242	1.241210	-0.356975
C5	-0.733242	-1.241210	-0.356975
C6	-0.958999	1.251749	-1.862456
C7	-0.958999	-1.251749	-1.862456
C8	-1.707591	0.000000	-2.304642
H9	-1.681333	1.239854	0.182228
H10	-0.137043	2.088146	-0.021308
H11	-1.681333	-1.239854	0.182228
H12	-0.137043	-2.088146	-0.021308
H13	-1.513416	2.154933	-2.112223
H14	0.004056	1.317495	-2.373438
H15	-1.513416	-2.154933	-2.112223
H16	0.004056	-1.317495	-2.373438
H17	-2.710901	0.000000	-1.873162
H18	-1.826517	0.000000	-3.385951
H19	0.950491	0.000000	-0.254172

***Z*-NNO(H)$^+$** E_h = -381.02078800 (Hartree)

N1	0.017441	0.006385	-0.008011
N2	0.001692	0.010858	1.249318
O3	1.148911	0.006700	1.896407
C4	-1.314760	0.078262	-0.658222
C5	1.174647	0.052212	-0.928436
C6	-1.391291	1.372259	-1.455091
C7	1.102019	1.357091	-1.718868
C8	-0.234690	1.475620	-2.441539
H9	-1.383040	-0.798227	-1.301718
H10	-2.052209	0.012167	0.135667
H11	1.062012	-0.817295	-1.575289
H12	2.117601	-0.052705	-0.396942
H13	-2.350441	1.386316	-1.969782
H14	-1.382241	2.216356	-0.763575
H15	1.935136	1.360275	-2.419732
H16	1.245604	2.196301	-1.036202
H17	-0.315917	0.686966	-3.192599
H18	-0.279911	2.424876	-2.970761
H19	1.914954	-0.009024	1.292686

***E*-NNO(H)$^+$** E_h = -381.03877873 (Hartree)

N1	-0.000150	0.005769	0.009011
N2	-0.001216	-0.011020	1.259594
O3	1.235490	-0.020911	1.762729
C4	-1.325206	0.080677	-0.644110
C5	1.184650	0.059693	-0.877210
C6	-1.388493	1.369689	-1.452108
C7	1.107230	1.355545	-1.680391
C8	-0.218478	1.462596	-2.423992
H9	-1.392612	-0.797937	-1.284891
H10	-2.071770	0.022975	0.142141
H11	1.095373	-0.812862	-1.524005
H12	2.080219	-0.021257	-0.274769
H13	-2.342113	1.381774	-1.977077
H14	-1.384488	2.219299	-0.767213
H15	1.950332	1.359337	-2.369056
H16	1.237352	2.201700	-1.003863
H17	-0.289674	0.664277	-3.165914
H18	-0.261462	2.405112	-2.965542
H19	1.092901	-0.045823	2.723466

NN(H)$^+$O E_h = -381.01901792 (Hartree)

N1	-0.005103	-0.003018	-0.044458
N2	0.115666	-0.036769	1.244497
O3	1.157824	-0.056691	1.868744
C4	-1.341235	0.102664	-0.652070
C5	1.186209	0.080894	-0.902385
C6	-1.404083	1.383533	-1.474437
C7	1.095199	1.368270	-1.714628
C8	-0.235915	1.455736	-2.452641
H9	-1.467272	-0.781458	-1.276427
H10	-2.085553	0.075974	0.144427
H11	1.155096	-0.799520	-1.544597
H12	2.061162	0.031777	-0.262172
H13	-2.358826	1.395060	-1.997543
H14	-1.388755	2.243218	-0.802460
H15	1.933259	1.377971	-2.409174
H16	1.217588	2.221857	-1.046080
H17	-0.310959	0.643098	-3.178699
H18	-0.286646	2.386885	-3.012643
H19	-0.783948	-0.055350	1.752324

Neutral *N*-nitrosopiperidine E_h = -380.68450736 (Hartree) -not included in Scheme 2-

N1	-0.001906	-0.023732	-0.003677
N2	-0.000773	0.046318	1.328545
O3	1.108594	0.086769	1.861100
C4	-1.288908	0.069098	-0.662873
C5	1.196886	0.059171	-0.822538
C6	-1.352505	1.341227	-1.499132
C7	1.152106	1.334187	-1.656633
C8	-0.154349	1.419930	-2.440945
H9	-1.418602	-0.807429	-1.303060
H10	-2.047007	0.047424	0.116371
H11	1.227376	-0.820150	-1.469888
H12	2.046916	0.031796	-0.147112
H13	-2.288629	1.361542	-2.057427
H14	-1.348751	2.202047	-0.827496
H15	2.010552	1.355280	-2.328099
H16	1.236975	2.192545	-0.987439
H17	-0.201500	0.593241	-3.155811
H18	-0.188562	2.342814	-3.019445

N(H$^+$)NO; E_h = -381.02248587 (Hartree) **PCM model (Acetone)**

N1	-0.03564526	0.00000000	0.05074295
N2	0.00177558	0.00000000	1.76395009
O3	1.07787291	0.00000000	2.15931444
C4	-0.74396185	1.23635771	-0.34332176
C5	-0.74396185	-1.23635771	-0.34332176
C6	-0.95558543	1.24972316	-1.84903101
C7	-0.95558543	-1.24972316	-1.84903101
C8	-1.70291339	0.00000000	-2.29818175
H9	-1.69847902	1.22873690	0.18874404
H10	-0.14705966	2.08188962	-0.00181679
H11	-0.14705966	-2.08188962	-0.00181679
H12	-1.50770097	2.15521078	-2.10283229
H13	-1.50770097	-2.15521078	-2.10283229
H14	0.01570647	1.30497440	-2.34734288
H15	0.01570647	-1.30497440	-2.34734288
H17	-2.70660050	0.00000000	-1.86593332
H18	-1.81546899	0.00000000	-3.38150200
H19	0.97604210	0.00000000	-0.25141072
H9	-1.69847902	-1.22873690	0.18874404

CARTESIAN COORDINATES in Å of the CAM-B3LYP/def2-TZVPP optimized geometries represented in Figure 1 in the main text.

TS0

N	-0.858315	-0.033010	0.576925
C	-1.261746	1.228141	-0.147390
C	-1.254918	-1.278855	-0.178393
C	-0.854163	1.245322	-1.607716
C	-0.847398	-1.257807	-1.638572
C	-1.342302	0.001261	-2.338232
H	-2.345227	1.237633	-0.028708
H	-0.860466	2.068364	0.416252
H	-2.338291	-1.296886	-0.059909
H	-0.848958	-2.130427	0.364524
H	-1.272426	2.148227	-2.051639
H	0.230507	1.333085	-1.691073
H	-1.261070	-2.151688	-2.104561
H	0.237694	-1.337781	-1.724241
H	-2.434272	-0.001241	-2.375867
H	-0.995254	0.014881	-3.369055
N	0.536816	-0.033187	0.812380
O	0.594304	-0.056166	2.019093
H1	-0.576763	-0.059180	2.072598

SD1

N	-0.737605	-0.033512	0.471052
C	-1.233818	1.233278	-0.107513
C	-1.223372	-1.288705	-0.140829
C	-0.849084	1.244779	-1.598291
C	-0.838942	-1.257256	-1.631410
C	-1.367263	0.000660	-2.298408
H	-2.312281	1.245326	0.037256
H	-0.776264	2.058866	0.432058
H	-2.301671	-1.313620	0.003532
H	-0.758841	-2.124452	0.376630
H	-1.278036	2.159193	-2.009227
H	0.233565	1.324678	-1.690450
H	-1.260699	-2.163876	-2.066397
H	0.244289	-1.326007	-1.725695
H	-2.459220	-0.003703	-2.304922
H	-1.048272	0.015836	-3.341448
N	0.668085	-0.031602	0.772659
O	0.407142	-0.048896	2.055344
H1	-0.859960	-0.050299	1.712475

CoIn

N	-0.458179687585	-0.001534483495	0.310533018334
N	0.258614615636	-0.003215912058	1.439226152730
O	1.592980239491	-0.003333545756	1.327158244161
C	-0.978331021849	1.235481701152	-0.182271479611
C	-0.979056996056	-1.236908603679	-0.185531791770
C	-0.885117703584	1.256202810176	-1.733470487810
C	-0.885635590185	-1.253739452073	-1.736741146047
C	-1.498857803600	0.002145742559	-2.326382275789
H	-2.042743553472	1.298404503951	0.083786384124
H	-0.447868244040	2.055914327935	0.291416086609
H	-2.043524815160	-1.299832697483	0.080323072849
H	-0.449304647506	-2.058915458850	0.286232424844
H	-1.398081296264	2.156396231227	-2.066427727587
H	0.164543956365	1.352671599511	-2.011902603344
H	-1.399121486653	-2.152793561191	-2.071971468776
H	0.163989603922	-1.350076175111	-2.015335788323
H	-2.578833908386	0.001996131650	-2.167295691285
H	-1.344627337430	0.003579709053	-3.406469327090
H	1.858705676359	-0.002445867518	0.389651403783

Table S1 compares the electronic excitation energies (gas and solution phases) obtained with two different approaches, that is, MS-CASPT2 and TD-DFT. In general, it is observed that the agreement between both theories is satisfactory.

Table S1. Comparison of MS-CASPT2 and TD-DFT vertical excitation energies (state-specific) in eV (nm) of *N*-nitrosopiperidines.[a]

Species	State	*gas*(PT2)	*gas*(DFT)	*ace*(PT2)	*ace*(DFT)
***E*-NNOH^{+}**[b]	S_1	5.56 (223)	5.32 (233)	5.59 (222)	5.40 (230)
	S_2	6.48	6.18	6.40	6.30
	S_3	8.81	6.33	8.86	6.33
**Z-NNOH^{+}**[b]	S_1	5.83 (213)	5.56 (223)	5.88 (211)	5.70 (218)
	S_2	6.47	6.28	6.48	6.19
	S_3	8.14	6.48	8.19	6.27
**NN(H^{+})O**[b]	S_1	4.19 (296)	4.39 (283)	4.31 (288)	4.58 (270)
	S_2	5.62	5.64	5.64	5.79
	S_3	8.93	6.04	9.10	5.99
**N(H^{+})NO**[b]	S_1	2.70 (459)	2.78 (446)	2.63 (471)	2.79 (445)
	S_2	6.54	5.76	6.60	5.53
	S_3	8.06	5.90	8.03	5.58
**NNO**[c]	S_1	3.41 (459)	3.45 (359)	3.53 (351)	3.69 (336)
	S_2	5.43	6.12	5.33	6.03
	S_3	7.55	7.44	7.65	7.47

[a]TD-DFT: CAM-B3LYP/def2-TZVPP.
[b]Reference wave function 1: SA2-CASSCF(16,14)/ANO-RCC (**C,N,O**[4s3p2d1f]/**H**[3s2p1d]).
[c]Reference wave function 2: SA2-CASSCF(16,13)/ANO-RCC (**C,N,O**[4s3p2d1f]/**H**[3s2p1d]).

CARTESIAN COORDINATES in Å of the MP2/def2-TZVPP optimized geometries of the X-H$^+$ complexes represented in Figure 2 in the main text.

Figure 2a

N	0.169127000000	0.055915000000	0.015110000000
N	0.290570000000	-0.024961000000	1.246590000000
O	1.513832000000	-0.031891000000	1.665177000000
C	-1.202659000000	0.071318000000	-0.518660000000
C	1.270215000000	0.165429000000	-0.959273000000
C	-1.407787000000	1.312381000000	-1.372218000000
C	1.056461000000	1.404829000000	-1.819979000000
C	-0.332286000000	1.428338000000	-2.444175000000
H	-1.309939000000	-0.835498000000	-1.113223000000
H	-1.875221000000	0.023926000000	0.330976000000
H	1.223730000000	-0.743348000000	-1.558845000000
H	2.208366000000	0.188561000000	-0.422349000000
H	-2.399481000000	1.248482000000	-1.816625000000
H	-1.397086000000	2.194051000000	-0.729472000000
H	1.830636000000	1.406547000000	-2.585209000000
H	1.208196000000	2.293561000000	-1.205669000000
H	-0.430590000000	0.602975000000	-3.152903000000
H	-0.467751000000	2.349609000000	-3.008064000000
H	1.464692000000	-0.097193000000	2.687236000000
O	1.480967000000	-0.197980000000	4.185201000000
H	1.133028000000	0.562015000000	4.663342000000
H	1.054901000000	-0.976205000000	4.559485000000

Figure 2b

N	0.139336000000	0.063514000000	0.011688000000
N	0.367459000000	-0.108541000000	1.253136000000
O	1.467550000000	-0.264674000000	1.730675000000
C	-1.244898000000	0.211414000000	-0.455925000000
C	1.228537000000	0.097901000000	-0.965600000000
C	-1.363504000000	1.454308000000	-1.323301000000
C	1.085018000000	1.333478000000	-1.845087000000
C	-0.316401000000	1.453430000000	-2.429425000000
H	-1.479284000000	-0.687258000000	-1.026531000000
H	-1.896081000000	0.250364000000	0.412459000000
H	1.145634000000	-0.817565000000	-1.552883000000
H	2.165633000000	0.080308000000	-0.422494000000
H	-2.370428000000	1.474803000000	-1.736392000000
H	-1.249930000000	2.340898000000	-0.697852000000
H	1.833195000000	1.263706000000	-2.632615000000
H	1.319920000000	2.219304000000	-1.253288000000
H	-0.504859000000	0.621591000000	-3.112166000000
H	-0.395092000000	2.368269000000	-3.014091000000
H	-0.488170000000	-0.117591000000	1.918057000000
O	-1.526488000000	-0.187291000000	3.077287000000
H	-2.007667000000	0.609400000000	3.323965000000
H	-2.161020000000	-0.911388000000	3.090435000000

Figure 2c

N	-0.098382000000	0.005456000000	0.118557000000
N	-0.137147000000	-0.026033000000	1.738640000000
O	0.893750000000	0.162812000000	2.185114000000
C	-0.818143000000	1.238852000000	-0.288283000000
C	-0.752707000000	-1.243025000000	-0.342486000000
C	-0.989637000000	1.275986000000	-1.797613000000
C	-0.931611000000	-1.225095000000	-1.851858000000
C	-1.681733000000	0.019286000000	-2.307607000000
H	-1.783030000000	1.221963000000	0.218181000000
H	-1.713770000000	-1.298000000000	0.167546000000
H	-0.244256000000	2.087426000000	0.074434000000
H	-0.133963000000	-2.075429000000	-0.017523000000
H	-1.562729000000	2.168589000000	-2.042810000000
H	-1.467148000000	-2.130908000000	-2.131108000000
H	-0.011426000000	1.385168000000	-2.269062000000
H	0.046047000000	-1.273013000000	-2.334562000000
H	-2.706984000000	-0.012410000000	-1.932363000000
H	-1.740736000000	0.041684000000	-3.394303000000
H	0.909124000000	0.040282000000	-0.154537000000
O	2.600773000000	0.008382000000	-0.569579000000
H	3.161132000000	0.704147000000	-0.212366000000
H	2.843934000000	-0.076117000000	-1.496590000000

Figure 2d

N	0.136143000000	-0.287662000000	-0.032738000000
N	0.055412000000	-0.615949000000	1.212817000000
O	1.090781000000	-0.394635000000	1.900337000000
C	-1.060237000000	-0.472631000000	-0.845430000000
C	1.288357000000	0.355144000000	-0.657600000000
C	-1.505249000000	0.878453000000	-1.389596000000
C	0.851821000000	1.713934000000	-1.195593000000
C	-0.356129000000	1.570381000000	-2.115884000000
H	-0.800847000000	-1.149878000000	-1.660101000000
H	-1.808534000000	-0.939081000000	-0.211568000000
H	1.612468000000	-0.297383000000	-1.469119000000
H	2.074449000000	0.427575000000	0.084913000000
H	-2.351551000000	0.720736000000	-2.056650000000
H	-1.846696000000	1.497303000000	-0.558066000000
H	1.694278000000	2.160367000000	-1.721839000000
H	0.604275000000	2.362791000000	-0.353836000000
H	-0.074904000000	0.983779000000	-2.993873000000
H	-0.673810000000	2.549982000000	-2.469840000000
H	0.732120000000	-0.761882000000	3.275166000000
O	0.514043000000	-1.031917000000	4.254934000000
S	-0.137354000000	0.148915000000	5.026899000000
O	-1.028976000000	0.872696000000	4.154808000000
O	-0.657336000000	-0.393377000000	6.253728000000
C	1.228110000000	1.189358000000	5.401590000000
H	0.845400000000	2.038774000000	5.959460000000
H	1.938901000000	0.627457000000	5.998043000000
H	1.676473000000	1.519572000000	4.469494000000

Figure 2e

N	0.552574000000	0.336175000000	0.105658000000
N	1.051007000000	0.702506000000	1.257455000000
O	2.258979000000	0.976154000000	1.308152000000
C	-0.879047000000	0.086965000000	0.027417000000
C	1.324411000000	0.286475000000	-1.128455000000
C	-1.504332000000	1.050897000000	-0.972988000000
C	0.711382000000	1.257939000000	-2.131325000000
C	-0.777514000000	0.979135000000	-2.312302000000
H	-1.020676000000	-0.945967000000	-0.295023000000
H	-1.292571000000	0.206198000000	1.025099000000
H	1.272049000000	-0.737781000000	-1.500717000000
H	2.352947000000	0.527568000000	-0.882632000000
H	-2.558124000000	0.799465000000	-1.085946000000
H	-1.443674000000	2.063775000000	-0.570912000000
H	1.244628000000	1.161641000000	-3.076152000000
H	0.857515000000	2.277117000000	-1.769363000000
H	-0.909832000000	-0.015979000000	-2.744365000000
H	-1.209996000000	1.694379000000	-3.010451000000
H	0.219544000000	0.811056000000	2.675522000000
O	-0.252122000000	0.958763000000	3.563988000000
S	-0.057771000000	-0.273198000000	4.508973000000
O	1.149429000000	-0.968067000000	4.141879000000
O	-0.212214000000	0.210746000000	5.852669000000
C	-1.434605000000	-1.291243000000	4.120358000000
H	-1.385513000000	-2.158715000000	4.771914000000
H	-2.343586000000	-0.727633000000	4.302035000000
H	-1.363646000000	-1.595344000000	3.081032000000

Figure 2f

N	0.007227000000	-0.469927000000	0.122301000000
N	0.034692000000	-0.896110000000	1.925281000000
O	-0.418828000000	-0.059232000000	2.539786000000
C	-0.435551000000	0.908200000000	-0.002961000000
C	-0.859319000000	-1.458765000000	-0.522014000000
C	-0.549954000000	1.275801000000	-1.480790000000
C	-0.965290000000	-1.139302000000	-2.009418000000
C	-1.457334000000	0.290269000000	-2.209558000000
H	-1.408868000000	0.999657000000	0.486339000000
H	-1.843661000000	-1.407980000000	-0.051824000000
H	0.281698000000	1.538821000000	0.519755000000
H	-0.434513000000	-2.445216000000	-0.345767000000
H	-0.932090000000	2.293150000000	-1.556204000000
H	-1.640907000000	-1.857215000000	-2.473216000000
H	0.447283000000	1.262780000000	-1.924316000000

H	0.016758000000	-1.264018000000	-2.469597000000
H	-2.475869000000	0.381053000000	-1.824402000000
H	-1.490620000000	0.529740000000	-3.271489000000
H	1.019067000000	-0.592873000000	-0.117329000000
O	2.659293000000	-0.686050000000	-0.451374000000
S	3.321013000000	-0.002446000000	0.684730000000
O	2.347288000000	0.337763000000	1.730378000000
O	4.497835000000	-0.709026000000	1.167941000000
C	3.891106000000	1.537773000000	0.028728000000
H	4.371256000000	2.095135000000	0.827393000000
H	4.600910000000	1.332089000000	-0.766719000000
H	3.038871000000	2.088455000000	-0.358311000000

Figure 2g

N	-0.675943000000	-0.149671000000	0.230877000000
N	-1.349587000000	0.126563000000	1.945970000000
O	-1.637191000000	1.212652000000	2.086690000000
C	-0.707328000000	1.127658000000	-0.464104000000
C	-1.511230000000	-1.196790000000	-0.361846000000
C	-0.260751000000	0.932607000000	-1.911452000000
C	-1.063867000000	-1.439790000000	-1.798898000000
C	-1.104803000000	-0.138898000000	-2.593832000000
H	-1.731547000000	1.506796000000	-0.428578000000
H	-2.545671000000	-0.847772000000	-0.340540000000
H	-0.056108000000	1.820549000000	0.066490000000
H	-1.415625000000	-2.083354000000	0.258628000000
H	-0.343004000000	1.887526000000	-2.429348000000
H	-1.713788000000	-2.193281000000	-2.242663000000
H	0.789706000000	0.639713000000	-1.912574000000
H	-0.048907000000	-1.842427000000	-1.791434000000
H	-2.138998000000	0.205228000000	-2.671822000000
H	-0.742426000000	-0.306349000000	-3.607190000000
H	0.294836000000	-0.444312000000	0.422947000000
O	2.122712000000	-0.080261000000	0.371973000000
S	2.477551000000	0.282772000000	1.747173000000
O	1.248948000000	0.482528000000	2.572096000000
O	3.427937000000	-0.614398000000	2.384718000000
C	3.224439000000	1.877255000000	1.669837000000
H	3.479265000000	2.186435000000	2.678565000000
H	4.118452000000	1.798979000000	1.058629000000
H	2.519263000000	2.571417000000	1.223599000000
H	0.885099000000	-0.680745000000	3.457863000000
O	0.546855000000	-1.433308000000	4.069044000000
S	0.493963000000	-2.783553000000	3.292983000000
O	-0.458763000000	-3.619203000000	3.972962000000
O	0.302467000000	-2.517248000000	1.885268000000
C	2.102610000000	-3.444047000000	3.517195000000
H	2.268969000000	-3.586176000000	4.580063000000
H	2.136130000000	-4.394731000000	2.992935000000
H	2.810858000000	-2.736053000000	3.095900000000
